# Successful medical management of acute mesenteric ischemia due to superior mesenteric and portal vein thrombosis in a 27-year-old man with protein S deficiency: a case report

**DOI:** 10.1186/s13256-017-1463-4

**Published:** 2017-11-09

**Authors:** N. P. Osti, D. N. Sah, R. S. Bhandari

**Affiliations:** 1 Maharajgunj Medical Campus, Institute of Medicine, Tribhuvan University Teaching Hospital, Kathmandu, Nepal; 2 Department of GI Surgery, Institute of Medicine, Tribhuvan University Teaching Hospital, Kathmandu, Nepal

**Keywords:** Acute mesenteric ischemia, Portomesenteric vein thrombosis, Protein S deficiency, Prothrombotic disease, Thromboprophylaxis, Medical management

## Abstract

**Background:**

Acute mesenteric ischemia poses a diagnostic challenge due to nonspecific clinical clues and lack of awareness owing to its rarity. Ischemia due to mesenteric venous thrombosis has a good prognosis compared to arterial cause and can be managed conservatively with early diagnosis. The portomesenteric venous system is an unusual site of thrombosis in patients with protein S deficiency, and its thrombosis is an uncommon cause of acute mesenteric ischemia.

**Case presentation:**

We present a case of a 27-year-old Mongolian man who presented with acute abdominal pain increasing in severity, and refractory to repeated attempts at treatment with a misdiagnosis of acute peptic ulcer disease. Contrast-enhanced computed tomography of his abdomen detected complete occlusion of the superior mesenteric vein, an extension of acute thrombus into the portal vein, and ischemic mid-jejunal loops. Early diagnosis and immediate anticoagulation with continuous intravenous infusion of unfractionated heparin prevented subsequent consequences. On further workup, our patient was diagnosed with isolated protein S deficiency. We started lifelong thromboprophylaxis with warfarin to prevent recurrence and our patient was asymptomatic on the latest follow-up 5 months after discharge.

**Conclusion:**

Despite accurate detection of acute mesenteric ischemia by contrast-enhanced computed tomography, high index of suspicion is indispensable for its early diagnosis. Early diagnosis and immediate anticoagulation will prevent subsequent complications and need for surgical intervention. Young patients without known risk factors presenting with venous thrombosis in atypical sites should be investigated for prothrombotic diseases.

## Background

Acute mesenteric ischemia (AMI) is a rare [1] gastrointestinal emergency that requires prompt recognition and intervention to prevent subsequent bowel infarction, perforation, and peritonitis. Despite the impressive accuracy of contrast-enhanced computed tomography (CECT), early diagnosis of AMI is challenging due to nonspecific clinical clues and lack of awareness of AMI, whether in resourceful or resource-poor setting. Mesenteric artery occlusion, venous thrombosis, nonocclusive mesenteric ischemia, and other indeterminate etiologies result in AMI, whereas mesenteric vein thrombosis is an uncommon cause [1]. Causes of mesenteric vein thrombosis are prothrombotic disease, local cause, abdominal surgery, malignancy or idiopathic causes, and prothrombotic disease accounts for 5–7% of cases [1, 2]. Inherited risk factors of venous thromboembolism (VTE) in young patients without other known risk factors are: factor V Leiden mutation, prothrombin gene mutation, methylene tetrahydrofolate reductase mutation, and, less commonly, protein C deficiency, protein S deficiency, and hyperhomocysteinemia [3]. Protein S is a vitamin K-dependent anticoagulant; its deficiency commonly presents with recurrent deep vein thrombosis, thrombophlebitis, or pulmonary embolism, and its prevalence in patients with mesenteric vein thrombosis and in general population are 2.6% and 0.03–0.13% respectively [4, 5]. We present a case of acute mesenteric ischemia due to superior mesenteric and portal vein thrombosis in a 27-year-old man with protein S deficiency.

## Case presentation

**Fig. 1:**
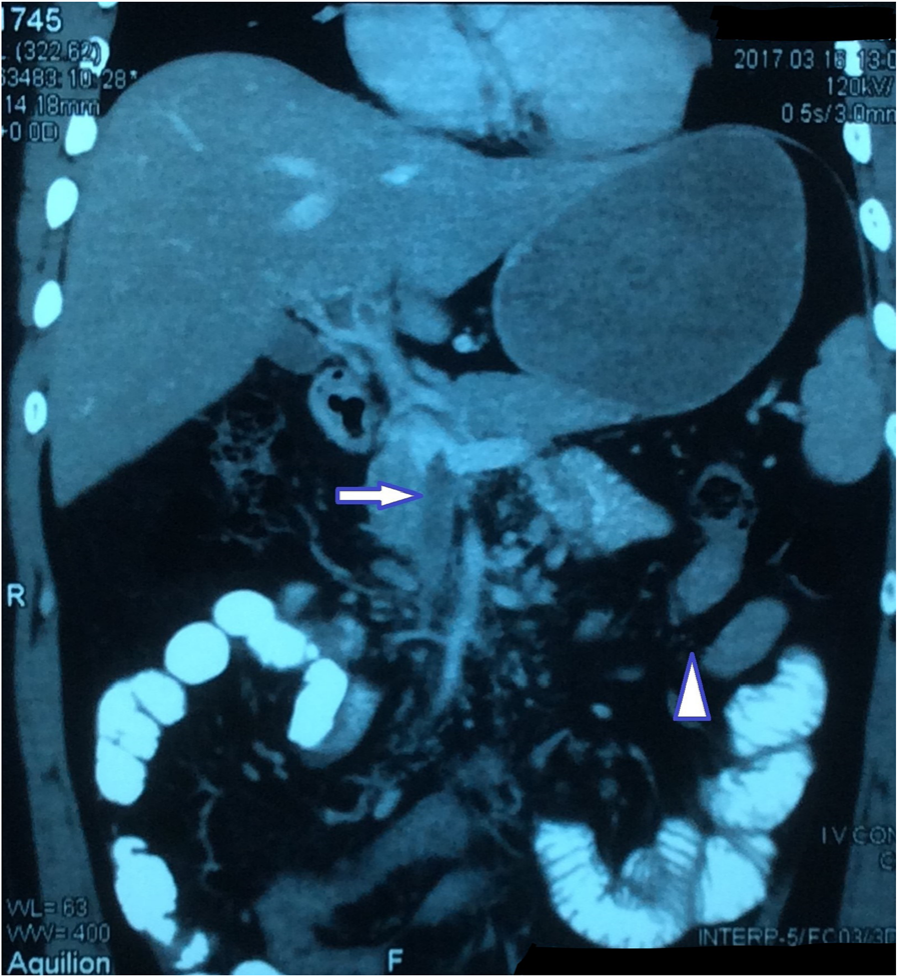
Contrast-enhanced computed tomography abdomen coronal sections. Complete occlusion of the superior mesenteric vein by thrombus (*white arrow*), nonenhanced jejunal loops (*arrowhead*)

**Fig. 2:**
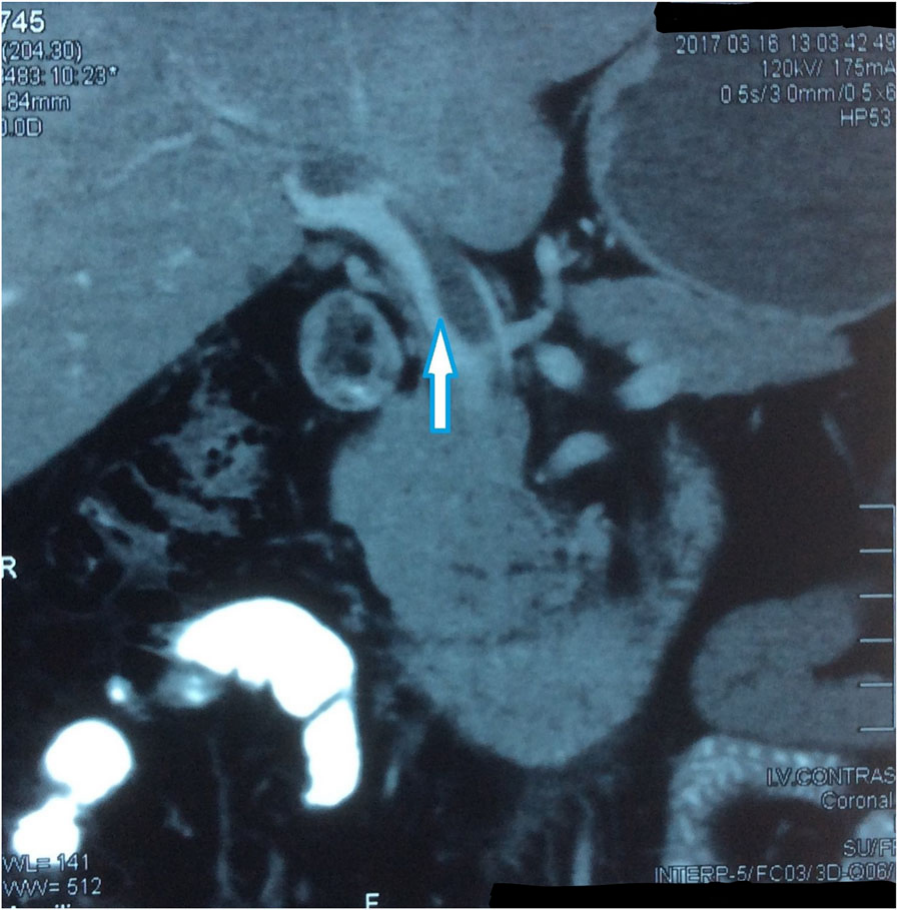
Contrast-enhanced computed tomography abdomen coronal sections. Thrombus in the portal vein (*white arrow*)

A 27-year-old Mongolian man from the central hilly region of Nepal presented with a 10-day history of pain in his left upper abdomen. Pain was acute in onset, crampy, nonradiating, and increasing in severity for the previous 3 days. He had had three episodes of vomiting during the last 2 days with no history of altered bowel habit or stool color. A systemic review was normal. He did not have significant past medical, surgical, or family history. Apparently, he had been managed at a local hospital in his home district with a diagnosis of acute peptic ulcer disease (APD) that did not relieve his symptoms, and he went to a teaching hospital in the capital. Again reassured it was APD and advised to follow up on an outpatient basis, he made frequent visits with similar complaints. Our patient’s symptoms increased in severity and he presented in the emergency department of the same hospital where investigations were carried out with normal hematological, biochemical, ultrasonography (USG), and upper gastrointestinal (UGI) endoscopy findings. With diagnostic uncertainty, an abdominal and pelvic CECT scan was done which revealed: complete occlusion of the superior mesenteric vein (Fig. 1, *white arrow*) and its tributaries due to acute thrombus extending into the main portal vein (Fig. 2, *white arrow*) narrowing its lumen; complete occlusion of the left portal vein and anterior division of the right portal vein without significant collaterals around its tributaries; a thickened edematous wall of 20 cm mid-jejunum (Fig. 1, *white arrowhead*), and ischemic changes to liver segments VII and VI. With the diagnosis of acute mesenteric ischemia secondary to portomesenteric vein thrombosis, our patient was referred to our hospital and we admitted him into the surgical intensive care unit for further management.

At admission, our patient was agitated but his vital signs were stable. His abdomen was soft with mild tenderness on the left hypochondrium and with normal bowel sounds. Other systemic examinations were normal. Routine investigations showed a white blood cell (WBC) count of 11,290/mm^3^ (N70), and a hemoglobin (Hb) level of 14.7 gm/dL, His platelet count was 366,000/mm^3^, and prothrombin time/international normalized ratio (PT/INR) was 16 sec/1.33. His liver function test, renal function test, and amylase level results were normal. Arterial blood gas analysis showed a lactate acid level of 0.5 mmol/L (ref, 0.7–2.5 mmol/L). Abdominal/pelvic X-rays showed no dilated bowel loops and no features of obstruction or pneumoperitoneum.

Owing to the young age of our patient, the unusual location, and absence of acquired risk factors of venous thrombosis, investigations to find possible prothrombotic disease were started. Clot-based coagulometer results showed plasma protein S activity of 17 s (ref. 77–143) and protein C activity of 114 s (ref. 67–195), whereas a chromogenic assay showed plasma antithrombin III activity of 75 s (ref. 70–122). A final diagnosis of acute mesenteric ischemia following acute superior mesenteric and portal vein thrombosis due to protein S deficiency was made.

On admission, we immediately started anticoagulation therapy with intravenous infusion of unfractioned heparin (UFH) monitored 6 hourly with activated partial thromboplastin time (aPTT). The therapeutic target range of 60–80 s of aPTT was achieved by the second day. We kept our patient on nil by mouth, prophylactic intravenous antibiotics, regular monitoring of his vital signs and routine investigations. Plasma lactate level and clinical features were used as markers for progression to extensive bowel ischemia and bowel infarction, which were maintained within normal limits. During the course of treatment, our patient developed episodes of malena for 4 days. Our patient’s symptoms gradually improved, he started tolerating an oral diet and developed no other significant issues. Warfarin bridging with heparin for 2 days with a target INR of 2–3 was done. We discharged our patient after 10 days with warfarin 5 mg taken orally twice daily, advised him to avoid exertion, any physical trauma, and strenuous work, and to follow up with a PT/INR report. After 2 weeks and subsequent 5-monthly follow-ups, our patient was comfortable, had normal bowel function, and maintained an INR of 2–3. We advised him on lifelong anticoagulation therapy with warfarin and regular PT/INR monitoring.

## Discussion

AMI poses a diagnostic difficulty in patients without known risk factors. Portomesenteric vein thrombosis presents with nonspecific signs and symptoms. CECT is the imaging of choice [6], but no promising diagnostic modality is available in resource-poor setting though abdominal X-ray and Doppler sonography could be helpful despite their own limitations. Often its diagnosis is missed and it gets managed in line with other abdominal conditions like APD, as in our case.

Nepal is a multiethnic country so variation in prevalence of protein S deficiency can be expected, as evidenced by a variable prevalence in Chinese, Japanese and Caucasian population, more so in Mongolian race [7]. The 2012 NICE guideline does not recommend routine testing of hereditary thrombophilia for asymptomatic relatives as a family history of VTE itself poses a risk of thromboembolism, even in the absence of identifiable hereditary thrombophilia [8]. Either a confirmatory second test on the index patient or testing on an asymptomatic relative would not affect the management and prophylaxis, but would be important for academic purposes.

Venous thrombosis causing AMI has a good prognosis than that due to an arterial cause. Kumar *et al*. in their retrospective study divide mesenteric venous thrombosis (MVT) into large vessel and small vessel thrombosis: large vessel thrombus extends into the portal or splenic vein and has lower risk of progression to bowel infarction; and small vessel thrombus involve isolated mesenteric veins and is more likely to be associated with prothrombotic disease [2]. But our patient with prothrombotic disease had thrombosis of the superior mesenteric vein extending up to the portal vein and its branches.

Management of portomesenteric vein thrombosis is concerned to prevent thrombus extension at an early stage and recurrence at the later stage. Acute phase management addresses resuscitation, antibiotic prophylaxis, and anticoagulation with heparin [unfractionated (UFH) or low-molecular-weight heparin (LMWH)] followed by warfarin targeting an INR of 2–3 [9]. Though LMWH is preferable over UFH due to its better safety profile, ease of administration, and not requiring regular lab monitoring; we used UFH infusion as we were concerned about the need of possible laparotomy during the initial days and continued later due to cost issues to the patient. Surgical management is guided by clinical findings of progression to bowel wall infarction perforation and peritonitis. Advancements in imaging, better understanding of the disease, and early detection of acute MVT has reduced the need for surgical intervention and fatal consequences [10]. Protein S deficiency increases the risk of recurrence in the index patient and the risk of VTE in first-degree relatives. Long-term prophylaxis for recurrent thrombosis is recommended for 6 months, but lifelong prophylaxis in cases where thrombophilia has been identified after the first event of thromboembolism, although this recommendation is not based on evidence [11]. Thromboprophylaxis in high-risk situations for both the index patient and first-degree relatives should be considered [8, 9, 11].

## Conclusion

AMI is a difficult clinical diagnosis; so a high index of suspicion and availability of advanced imaging facility is indispensable. Abdominal pain out of proportion to physical findings and not explained by common acute abdominal conditions should raise suspicion of AMI. Awareness of AMI among primary health-care physicians will help early referral to higher centers and prevent unfavorable consequences. Venous thrombosis in young patients at uncommon sites and with no known risk factors is likely to have underlying prothrombotic disease; it will influence the duration of thromboprophylaxis.

## Abbreviations

AMI: Acute mesenteric ischemia
APD: Acute peptic ulcer disease
aPTT: Activated partial thromboplastin time
CECT: Contrast-enhanced computed tomography
LMWH: Low-molecular-weight heparin
MVT: Mesenteric venous thrombosis
PT/INR: Prothrombin time/international normalized ratio
UFH: Unfractionated heparin
VTE: Venous thromboembolism
